# SUMOylation of Syntaxin1A regulates presynaptic endocytosis

**DOI:** 10.1038/srep17669

**Published:** 2015-12-04

**Authors:** Tim J. Craig, Dina Anderson, Ashley J. Evans, Fatima Girach, Jeremy M. Henley

**Affiliations:** 1 School of Biochemistry, Medical Sciences Building, University of Bristol, University Walk, Bristol, BS8 1TD, U.K

## Abstract

Neurotransmitter release from the presynaptic terminal is under very precise spatial and temporal control. Following neurotransmitter release, synaptic vesicles are recycled by endocytosis and refilled with neurotransmitter. During the exocytosis event leading to release, SNARE proteins provide most of the mechanical force for membrane fusion. Here, we show one of these proteins, Syntaxin1A, is SUMOylated near its C-terminal transmembrane domain in an activity-dependent manner. Preventing SUMOylation of Syntaxin1A reduces its interaction with other SNARE proteins and disrupts the balance of synaptic vesicle endo/exocytosis, resulting in an increase in endocytosis. These results indicate that SUMOylation regulates the emerging role of Syntaxin1A in vesicle endocytosis, which in turn, modulates neurotransmitter release and synaptic function.

The primary function of the presynapse is the timely release of neurotransmitter via fusion of synaptic vesicles (SVs) with the presynaptic membrane^1^. The process of SV exocytosis is exquisitely regulated involving many different proteins^2^. Presynaptic SNARE (soluble N-ethylmaleimide sensitive factor attachment protein receptors) proteins, Syntaxin1 (Stx1), SNAP-25 and VAMP-2/synaptobrevin-2 are fundamental to neurotransmitter release. Syntaxin1 and SNAP-25^3^ are anchored to the presynaptic membrane, whereas VAMP-2 is located on the SV membrane. These three proteins form a four-helix bundle SNARE complex (in which 2 helices come from SNAP-25 and 1 each from VAMP-2 and Stx1), which provides most of the mechanical force for membrane fusion to occur^4^. Additional to the SNARE complex, many other proteins are vital for accurate SV exocytosis, including Munc18^5^, Munc13^6^, synaptotagmin^7^ (the calcium sensor), and RIM proteins^8^,^9^. Nonetheless, despite remarkable progress in elucidating many of the core mechanisms of activity dependent neurotransmitter release there remain many unanswered questions. Particularly intriguing are recent reports indicating that, in addition to their vital roles in exocytosis, SNARE proteins are also required for synaptic vesicle endocytosis^10^,^11^.

SUMOylation is a post-translational modification of proteins, analogous to ubiquitination. The Small Ubiquitin-like MOdifier (SUMO) is conjugated to target proteins, via an isopeptide bond to the primary amine groups of lysine residues (for reviews see^12^,^13^). Critical enzymes in the process are Ubc9, the only SUMO E2 enzyme, which is necessary and sufficient for SUMO conjugation, and the SENP SUMO-specific proteases, which catalyse deconjugation. SUMOylation has been characterised most extensively in the nucleus, where it regulates a variety of processes including transcription and chromosome segregation. Over recent years, however, SUMOylation of synaptic proteins has been shown to play key roles in kainate receptor trafficking^14^, synapse formation^15^, and various forms of synaptic plasticity^16^,^17^,^18^. While presynaptic glutamate release is modulated by protein SUMOylation^19^, identifying the specific proteins that are SUMOylated *in vivo* and defining the functional consequences is a formidable task. Nonetheless, significant progress has been made, for example we recently identified RIM1 as a SUMO substrate and demonstrated that this SUMOylation regulates Ca^2+^ channel clustering at the presynaptic terminal, modulating activity dependent neurotransmitter release^20^. Intriguingly, it has also been reported that SUMOylation affects insulin release from pancreatic β-cells^21^, implying that this modification may have a general role in regulating many different secretory pathways.

Here, we report that another critically important presynaptic protein, Syntaxin1A (Stx1A), is an activity-dependent SUMO substrate. Preventing Stx1A SUMOylation causes defects in the synaptic vesicle cycle, leading to increased rates of vesicle endocytosis, indicating that SUMOylation of Stx1A is directly involved in the fine control of activity-dependent neurotransmitter release.

## Results and discussion

### Syntaxin1A is an activity-dependent SUMO substrate

**Figure 1:**
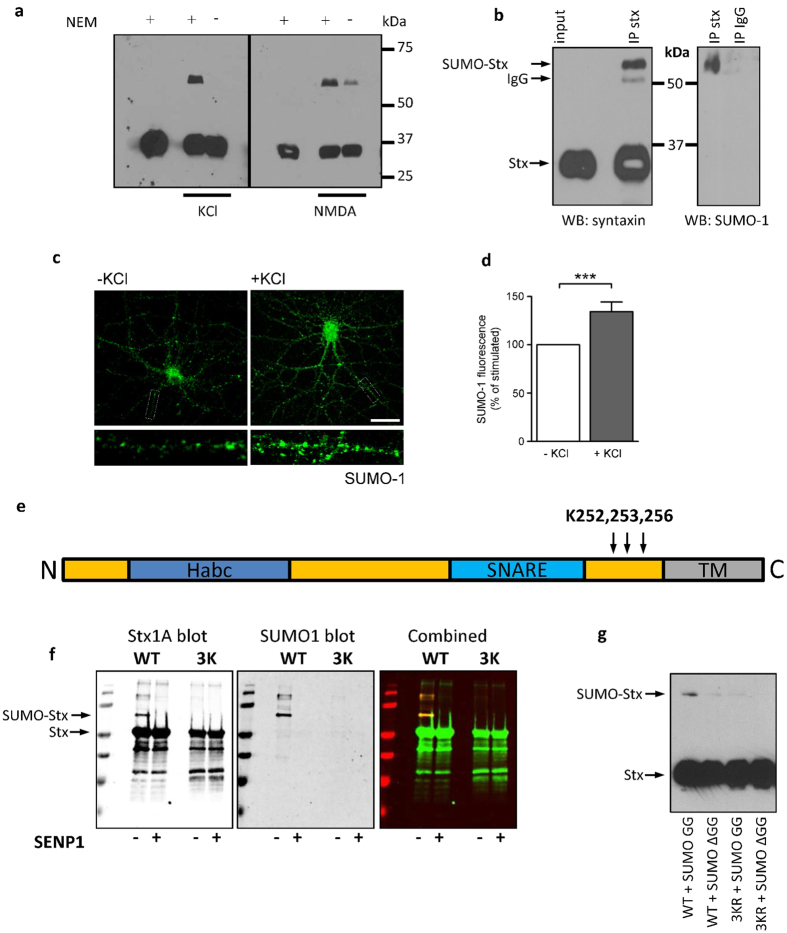
Syntaxin1A undergoes activity-dependent SUMOylation in neurones. (**a)** Syntaxin (HPC-1) Western blot of neuronal lysates. Neurones were treated with KCl (30 mM) or NMDA (50 μM) for 3 minutes followed by a 27 minute incubation in HBS. 20 mM NEM was included or omitted from lysis buffer as indicated. (**b)** Immunoprecipitation of Stx1A from neuronal lysates in **a**. Immunoprecipitated samples were blotted for either Stx1A or SUMO-1. (**c)** Immunostaining of hippocampal neurones for SUMO-1, either with or without 30 min 30 mM KCl stimulation. (**d)** Quantification of **c**. ***p < 0.001 on Student’s t-test. n = 35 (**e)** Schematic diagram of Stx1A with SUMOylation sites shown. (**f)** SUMOylation assay in *E.Coli* showing abrogation of SUMOylation in the 3KR mutant. WT or 3KR GST-Stx1A was transformed into BL21 *E.Coli* together with the minimal SUMOylation machinery. Bacteria were lysed and blotted for both Stx1A and SUMO-1. Samples were treated with SENP1 as indicated to confirm that the upper bands are SUMO-Stx1A. (**g)** SUMO assay in HEK293 cells showing loss of SUMOylation in the Stx1A 3KR mutant.

Stx1A was identified as potential synaptic SUMO substrate from a bioinformatics database search for proteins containing the consensus SUMOylation motif. A major hit was Stx1A, which possesses a single high probability SUMOylation site at K204. To validate that Stx1A is SUMOylated in brain we immunoblotted cortical neuronal lysates with anti-Stx1A antibody in the presence and absence of 20 mM N-ethylmaleimide (NEM), which blocks the catalytic activity of the deSUMOylating SENP enzymes to preserve the SUMOylation status of target proteins. Under basal, non-stimulated conditions we observed a single Stx1A band. However, following stimulation of the cortical neurones with either 30 mM KCl for 30 minutes or 50 μM NDMA for 3 minutes followed by 27 minutes without drug, a higher molecular weight band was observed (Fig. 1a). Note that although SUMO is a 10 kDa peptide, as observed for other SUMOylated proteins^14^,^20^, modification results in a >20 kDa band shift, owing to the branched nature of the modification causing the SUMOylated proteins to be retarded in the gel. Consistent with this higher Mr band being due to SUMO conjugation, it was dramatically decreased when NEM was omitted from the lysis buffer. We next immunoprecipitated Stx1A from brain lysate. The higher Mr band was immunoprecipitated by the Stx1A antibody and was also reactive for SUMO-1, again indicating that it is SUMOylated Stx1A (Fig. 1b). Furthermore, in agreement with a report that neuronal activation regulates the SUMOylation of pre- and postsynaptic proteins^19^, 30 mM KCl treatment caused a significant global increase in SUMO-1 immunoreactivity in neuronal processes (Fig. 1c,d). Taken together these data indicate that Stx1A is a substrate for activity-dependent SUMOylation.

To identify which lysines in Stx1A are SUMOylated, we performed site-directed mutagenesis. Stx1A contains only one high-probability consensus lysine residue (K204) predicted by SUMOsp^22^. However, mutation of K204 to a non-SUMOylatable arginine did not inhibit Stx1A SUMOylation in either a HEK293 cell or an *E.Coli* based assay (Fig. S1a,b). Like ubiquitin, SUMO can modify other lysine residues in addition to the lysine within the consensus sequence^23^. Therefore, we systematically mutated flanking lysine residues and discovered that SUMO-1 can conjugate at any one of three lysines (K252, K253 or K256) near the C-terminal transmembrane domain of Stx1A (Fig. 1e). Only mutation of all three lysines to arginines (3KR mutation) effectively prevented Stx1A SUMOylation in the *E.Coli* assay (Fig. S1c, Fig. 1f).

To confirm this result, we repeated this experiment in a HEK293 cell-based SUMO assay. Stx1A WT or 3KR were co-expressed with Ubc9 and YFP-SUMO-1 or YFP-SUMO-1ΔGG. Note that the di-glycine motif is essential for conjugation^12^, therefore SUMO-1ΔGG cannot be attached to substrate proteins. Expression of Stx1A WT with YFP-SUMO-GG and Ubc9 yielded a high molecular weight band on a Stx1A blot, consistent with Stx1A SUMOylation by YFP-SUMO. In stark contrast, there was little or no detectable higher Mr band respectively with co-expression of YFP-SUMO-ΔGG with Stx1A 3KR (Fig. 1g). These data confirm that K252, K253 and K256 are the only SUMOylatable residues in Stx1A.

### SUMOylation of Syntaxin1A regulates critical protein-protein interactions

Due to the position of the three mutated residues (between the SNARE motif and the transmembrane domain) it is possible that the mutation alone might influence the function of Stx1A, independent of any deficit in SUMOylation. To exclude this possibility we tested the binding and trafficking properties of this mutant. We observed no difference in SNAP-25 or Munc18a binding to WT or 3KR Stx1A, indicating that the SUMO-defective mutant Stx1A protein can interact with critical partners and presumably participate normally in SNARE complexes (Fig. S2a). Additionally, we did not detect any changes in neuronal 3KR Stx1A distribution compared to the wild-type protein (Fig. S2b).

**Figure 2:**
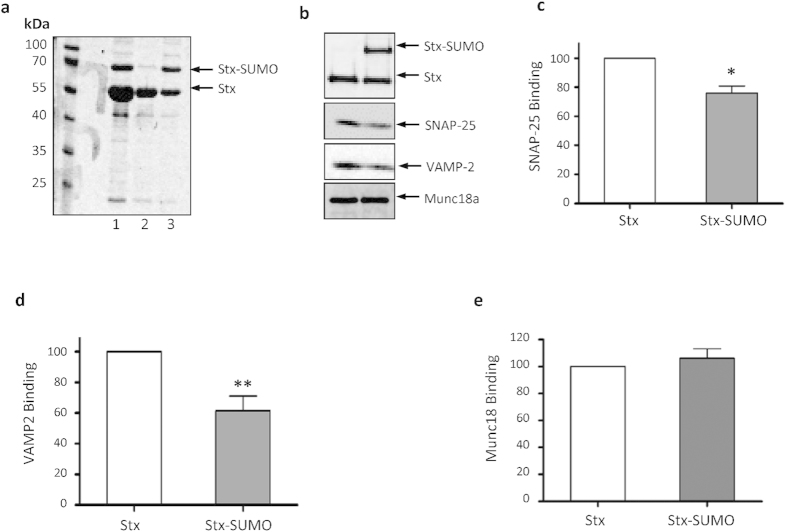
SUMOylation of Stx1A reduces binding to SNAP-25 and VAMP-2 but not Munc18a. (**a**) Coomassie stain of purification of SUMOylated Stx1A from BL21 *E.Coli*. GST-tagged Enhanced-SUMO Stx1A (GST-ES-Stx1A) was expressed in *E.Coli* together with minimal SUMOylation machinery and His_6_-tagged SUMO-1. Lane 1: Eluate from 1^st^ stage purification (glutathione beads), Lane 2: Flow through from second stage purification (Ni-NTA beads), Lane 3: Eluate from Ni-NTA beads. (**b)** Representative Western blots of affinity pulldowns from neuronal lysate with SUMOylated/SENP treated SUMOylated Stx1A. (**c)** Quantification of SNAP-25 binding to Stx1A/Stx1A-SUMO. *p < 0.05 (Student’s t-Test). n = 5. (**d)** Quantification of VAMP-2 binding to Stx1A/Stx1A-SUMO. **p < 0.01 (Student’s t-Test). n = 4. (**e)** Quantification of Munc18a binding to Stx1A/Stx1A-SUMO. n = 5.

It is also possible that these three lysines could be targets for ubiquitination as well as SUMOylation *in vivo*. To address this, we transfected HEK293T cells with both Stx1A WT and 3KR, and used the proteasome inhibitor MG132 to prevent degradation and enhance levels of ubiquitinated protein. We observed no change in high molecular weight species of either WT or 3KR Stx1A with MG132 treatment (Fig. S2c,d), even though global cellular ubiquitination was robustly increased (Fig. S2e). These data indicate that, in our experiments, Stx1A is not a detectable ubiquitination substrate in HEK cells. We note, however, that Stx1A has previously been reported to undergo ubiquitination^24^,^25^. Because it is not feasible to directly test 3KR ubiquitination in our neuronal systems, we cannot unequivocally state that the 3KR mutation does not affect ubiquitination of Stx1A in neurones. Nonetheless, removing ubiquitination sites would be expected to increase protein stability but recombinant WT and 3KR proteins are expressed at identical levels in neurones (Fig. S3a,b), consistent with the 3KR mutant having no effect on protein turnover. Thus, whilst we cannot formally rule out an effect on ubiquitination, the 3KR mutation seems to only affect SUMOylation of Stx1A, with no apparent effect on its binding to critical cellular partners or on Stx1A turnover time. To determine if SUMOylation causes changes in core Stx1A protein interactions, we performed binding assays using recombinantly SUMOylated Stx1A. The best characterised interactors with Stx1A are Munc18a and SNAP-25^26^. The interaction with SNAP-25 is required for SNARE complex formation and exocytosis, whereas Munc18a interacts in with Stx1A in two distinct ways; Mode 1) with Stx1A alone (in the ‘closed’ conformation) and Mode 2) with the assembled SNARE complex. Mode 1 is thought to be required for SNARE complex formation, and mode 2 is essential for exocytosis^27^. We performed pulldowns from cortical neuronal lysate using Stx1A SUMOylated in a bacterial system (Fig. 2a) and blotted for SNAP-25, Munc18a and VAMP-2. SUMOylated Stx1A binding to SNAP-25 and VAMP-2 was significantly decreased, but there was no change in binding to Munc18a (Fig. 2b–e). These data suggest that SUMOylation of Stx1A can perturb SNARE complex formation without altering the interaction of Stx1A with Munc18a, therefore favouring binding mode 1.

### Syntaxin1A SUMOylation regulates the synaptic vesicle cycle

**Figure 3:**
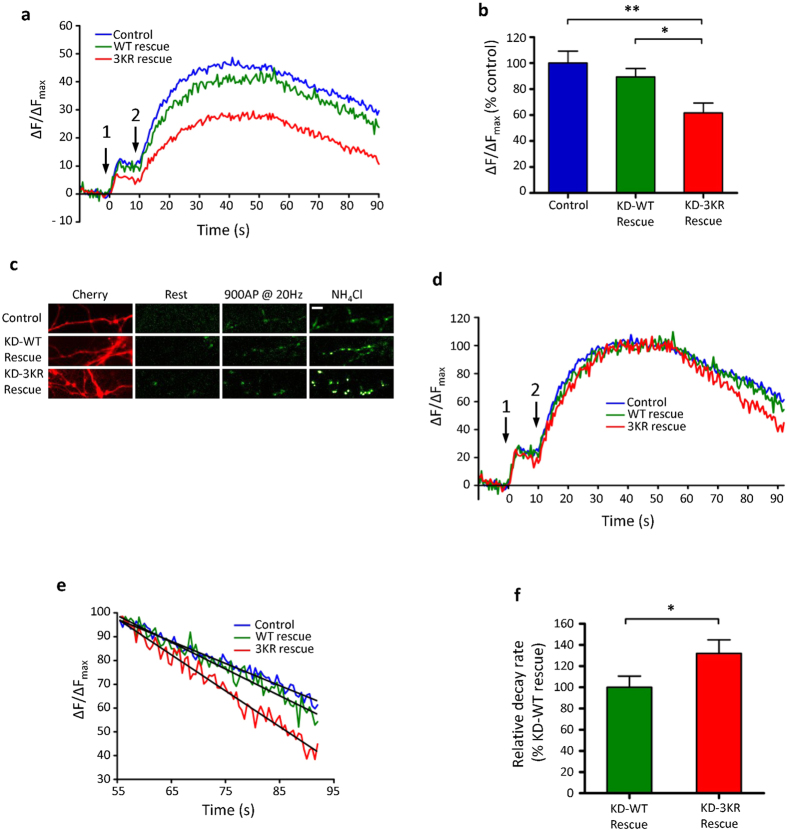
Stx1A SUMOylation regulates the synaptic vesicle cycle. (**a**) Exocytosis profile measured by vGlut-pHluorin in control (n = 9), WT-Rescue (n = 13) and 3KR-Rescue (n = 16) neurones. Stimulation 1 is 66 APs @ 33 Hz to release the RRP. Stimulation 2 is 900 APs @ 20 Hz to release the entire recycling pool. (**b)** Quantification of the peak evoked vGlut-pHluorin signal (taken as the signal directly after the second stimulation). *p < 0.05, **p < 0.01 (1 way ANOVA with Bonferroni’s post-hoc test). n numbers as above. (**c)** Representative images of experiment (**a)** Scale bar = 10 μm. (**d)** Data from experiment (**a**) normalised to the peak evoked signal (in order to measure exo- and endocytosis rates). (**e)** Linear regression fitting of the endocytosis profiles from experiment (**a,f**) Quantification of the rate of endocytosis from linear regression plots. *p < 0.05 (Student’s t-Test).

To define the effect of Stx1A SUMOylation on synaptic vesicle exocytosis we used shRNA to knockdown endogenous Stx1A and 1B with a previously validated sequence^28^ and concomitantly expressed an shRNA-resistant form of either wild-type (WT-rescue) or 3KR Stx1A (3KR-rescue). The shRNA and the replacement construct, together with a fluorescent marker (mCherry) were expressed on the same plasmid to ensure co-expression in the same cells. Using immunofluorescent staining of Stx1A in fixed hippocampal neurones, we established that the shRNA causes a ~50% reduction in Stx1A (Fig. S3a,b), and both WT and 3KR rescue constructs returned Stx1A expression to control levels. We next assessed the effects of replacing endogenous Stx1A with either WT or non-SUMOylatable 3KR on synaptic vesicle exocytosis using the pH-sensitive reporter vGlut-pHluorin to measure vesicle fusion^29^. This reporter consists of the vesicular Glutamate Transporter expressing a super-ecliptic pHluorin in its luminal domain. At the acidic pH of the vesicle lumen, fluorescence is quenched; however, upon exocytosis, the pHluorin is exposed to the neutral extracellular pH and fluoresces. Exocytosis was stimulated by two different protocols: 40 AP @ 20 Hz, followed by 900 AP @ 20 Hz. The extent of vesicle fusion was expressed as a percentage of total vGlut-pHluorin fluorescence defined by addition of NH_4_Cl which reversibly collapses intracellular pH gradients and allows visualisation of all pH-sensitive fluorophore. As shown in Fig. 3a, there were clear differences between WT and 3KR Stx1A. WT-rescue neurones displayed a similar exocytosis profile to control neurones, whereas 3KR-rescue neurones showed a significant ~40% decrease in maximal exocytosis levels compared to both WT-rescue and control cells (Fig. 3a–c). These data indicate that the 3KR mutation of syntaxin is unable to support normal synaptic vesicle exocytosis, presumably due to the absence of SUMOylation (although as previously stated we cannot formally rule out an effect of ubiquitination in this function). Note that because knock down of Stx1 without molecular replacement severely compromises cell viability we were unable to obtain any reliable exocytosis profiles from neurons expressing just Stx1shRNA.

To compare differences in signal decay rates after stimulation values were normalised to peak levels of exocytosis of each cell (Fig. 3d). We then measured the slope of the linear phase of the vGlut-pHluorin signal decay attributable to endocytosis and reacidification of synaptic vesicles (Fig. 3e). The decay rates of control and WT-rescue cells were very similar. The 3KR-rescue neurones, however, had a significantly faster rate of signal decay, indicating a faster rate of vesicle endocytosis (Fig. 3f). Because endocytosis occurs concurrently with exocytosis, these data suggest that the reduced levels of vGlut-pHluorin signal in the 3KR-rescue cells is due to an increase in the rate of vesicle endocytosis. We note that our endocytosis profiles are linear. We attribute this to the experimental temperature and robust stimulation protocol used^30^,^31^,^32^.

### Syntaxin1A SUMOylation is required for regulation of synaptic vesicle endocytosis

**Figure 4:**
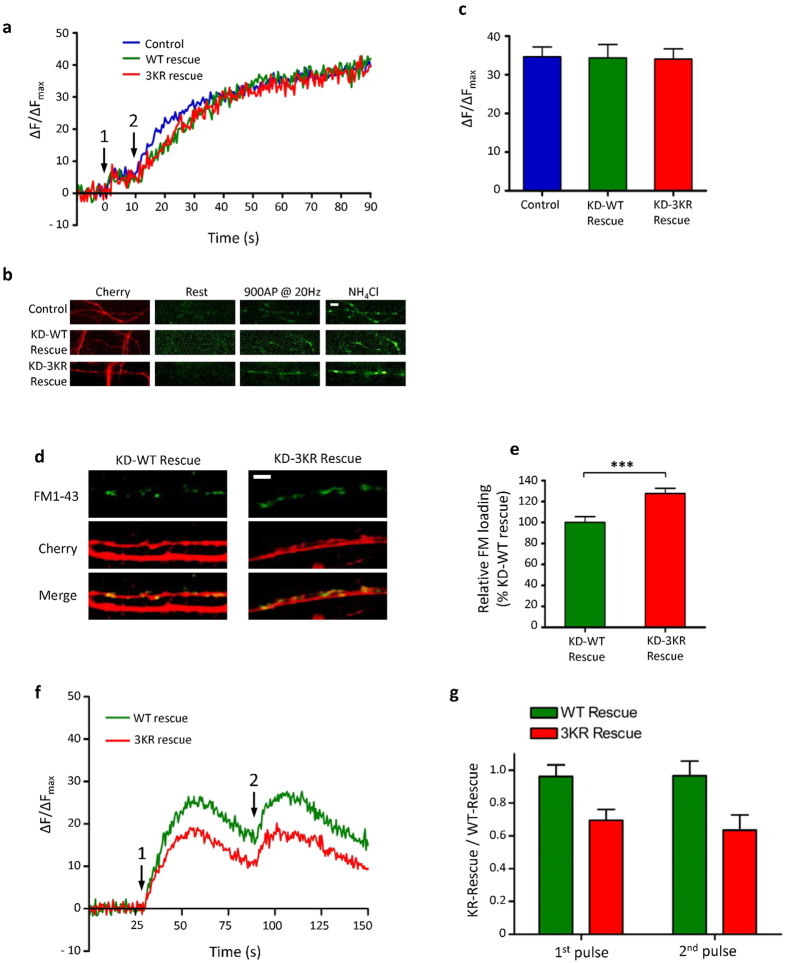
Syntaxin1A SUMOylation regulates the rate of synaptic vesicle endocytosis. (**a)** Exocytosis profile measured by vGlut-pHluorin in control (n = 10), WT-Rescue (n = 10) and 3KR-Rescue (n = 11) neurones, in the presence of 1 μM bafilomycin 1A. Stimulation 1 is 66 APs @ 33 Hz to release the RRP. Stimulation 2 is 900 APs @ 20 Hz to release the entire recycling pool. (**b)** Representative images showing vGlut-pHluorin fluorescence at the indicated timepoints for the different conditions, plus mCherry as a transfection marker. Scale bar = 10 μm. (**c)** Quantification of the peak evoked vGlut-pHluorin signal (taken as the signal directly after the second stimulation), showing no significant difference. n numbers as above. (**d)** Representative FM1-43 dye uptake images for WT-rescue (n = 22) and 3KR-rescue neurones (n = 25), elicited by 1 min incubation in 30 mM KCl. mCherry is used as a transfection marker. Scale bar = 10 μm. (**e)** Quantification of relative FM1-43 uptake (as percentage of WT-rescue neurones). ***p < 0.001 (Student’s T-test). (**f)** Exocytosis profile measured by vGlut-pHluorin in WT-Rescue and 3KR-Rescue neurones. Both stimulations indicated are 900 APs @ 33 Hz. (**g)** Quantification of exocytosis peaks (i.e. fluorescence level directly after stimulations). No significant differences were seen in these peaks between the first and second stimulations in either WT-rescue (n = 9) or 3KR-rescue (n = 11) neurones.

To further investigate the effects of Stx1A SUMOylation on endocytosis, we repeated the vGlut-pHluorin exocytosis assay in the presence of bafilomycin 1A, an inhibitor of the vesicular H^+^-ATPase. Under these conditions, vesicle reacidification following endocytosis is inhibited, and therefore neutral pH and SEP fluorescence is maintained after endocytosis. In this way the cumulative increase in fluorescence due to exocytosis can be monitored in the absence of any contribution from compensatory endocytosis. These experiments revealed no difference between the rate or levels of exocytosis in WT-rescue and 3KR-rescue cells (Fig. 4a–c), although both WT and 3KR rescue cells had a lower initial rate of exocytosis compared to the control. This is likely because the shRNA knocks down both Stx1A and 1B whereas the only Stx1A was replaced. Interestingly, and consistent with our results, another recent study has indicated a role for Syntaxin1B in fast vesicle exocytosis^33^. Due to our relatively modest level of endogenous Stx1 knockdown (~50%), we cannot formally discount that a more complete molecular replacement with 3KR Stx1 would result in a defect in exocytosis as well as endocytosis. However, it seems likely that our knockdown of Stx1 is sufficient for almost complete loss of presynaptic function, as we could not elicit any presynaptic responses from knockdown cells in the absence of rescue.

We next used a lipophillic FM-dye uptake assays to more directly measure vesicle endocytosis^34^. Intact cells are exposed to the dye, which stains the plasma membrane, and then depolarised with KCl, which causes accumulation in the presynaptic terminal via the exposure of exocytosed vesicular membrane and subsequent endocytosis. Importantly, as dye continues to be applied after the stimulus is removed, the dye loading can be used as a measure of the combination of exo- and endocytosis. Because we showed exocytosis is unchanged between WT- and 3KR-rescued neurones (Fig. 4a–c), we attribute differences in FM-dye uptake to an increase in endocytosis. We observed a robust increase in FM-dye uptake in 3KR-rescue cells compared to WT-rescue cells (Fig. 4d,e). This increase correlated well with the increases in the decay of the vGlut-pHluorin signal in the 3KR-rescue, again consistent with enhanced endocytosis under conditions of decreased Stx1A SUMOylation. Thus, although at first sight it may seem counter-intuitive, we attribute our results to mis-regulation of synaptic vesicle endocytosis, whereby there is a net increase in endocytosis resulting in the recovery of more membrane than was initially exocytosed.

This unexpected result indicates that SUMOylation of Stx1A acts as a regulating factor that coordinates the balance between endo- and exocytosis at the presynapse. We propose that this constitutes a ‘quality control’ step, to ensure that the necessary synaptic vesicle proteins are retrieved from the plasma membrane before endocytosis. This hypothesis would predict that the increased rate of endocytosis observed following inhibition of Stx1A SUMOylation would result in internalised vesicles that are not competent for further rounds of fusion, which is entirely consistent with the deficit in exocytosis shown in Fig. 3. However, we also acknowledge that there are other possible explanations. For example, similar results would be obtained if there was an increase in the recycling vesicle pool which is only mobilised by the strong KCl stimulus used in the FM1-43 experiments but not the field stimulation used in the vGLUT-pHluorin assays. We tested this possibility in two ways. Firstly, we analysed the total levels of vGLUT-pHluorin expression as revealed by NH_4_Cl treatment (which is indicative of synaptic vesicle number). (Fig. S4a) We found no significant differences in total vGLUT expression between WT-rescue and 3KR-rescue neurones. Secondly, we performed immunostaining for the synaptic vesicle markers VAMP2 and synaptophysin, the intensity of which is indicative of synaptic vesicle pool size (Fig. S4b,c). Again, we found no significant difference in VAMP2 staining between WT-rescue and 3KR-rescue neurones. However, there was a small (10%) but significant decrease in synaptophysin staining in 3KR-rescue neurones relative to WT-rescue neurones. This was unexpected, and may indicate defects in recycling of the synaptic vesicle contents during endocytosis caused by ablation of Stx1A SUMOylation. Overall, these data argue against a model in which the synaptic vesicle pool size is increased in 3KR-rescue neurones, suggesting that the increased FM1-43 loading seen in Fig. 4d,e is due to a change synaptic vesicle endocytosis.

**Figure 5:**
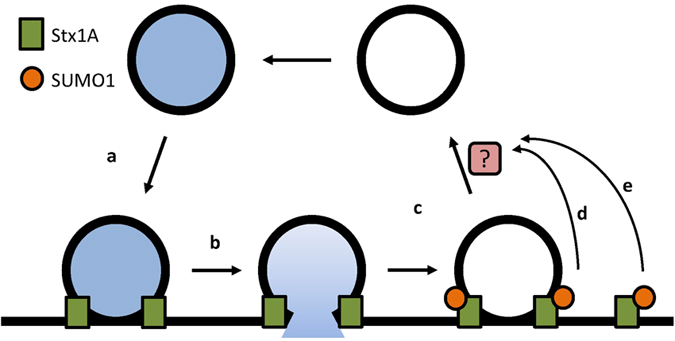
Model for the action of Syntaxin1A SUMOylation. Schematic of our proposed mechanism for the action of SUMOylated Stx1A on the synaptic vesicle cycle. We find no evidence that Stx1A SUMOylation is involved directly in synaptic vesicle docking (**a**) exocytosis (**b**). Instead it appears that SUMOylation acts as a regulator of vesicle endocytosis (**c**). Due to our data on the inhibition of SNARE interactions by SUMOylation, we propose that this is effected by a non-SNARE associated pool of Stx1A (**e**), rather than the same Stx1A pool which mediates exocytosis (**d**).

To further the effect of SUMOylation on synaptic vesicle recycling, we performed a modification of the vGlut-pHluorin assay, using two strong stimuli (900 Aps @ 33 Hz) interspersed with a 30 s of rest. We reasoned that the first stimulation would completely deplete the releasable vesicle pool, meaning that subsequent exocytosis evoked by the second stimulation would have to be mediated by recycled vesicles that had fused to and been endocytosed from the presynaptic membrane. We observed a significantly lower level of exocytosis in the 3KR-rescue cells (Fig. 4f,g), consistent with our results in Fig 3. However, there was no difference in the peak exocytosis level between the first and second stimulations for either the WT-rescue or the 3KR-rescue (Fig. 4f,g). There are two possible interpretations of these data: either the stimulation we used was insufficient to deplete the releasable vesicle pool, or these data indicate that vesicles were efficiently retrieved and recycled in both conditions. The first explanation seems unlikely, as we noted a plateauing of the vGlut-pHluorin signal prior to the end of each stimulation, indicating that this stimulation protocol is indeed saturating. Therefore it seems unlikely that the inhibition of SUMOylation of Stx1A inhibits synaptic vesicle recycling. Rather, our data favour a model in which Stx1A SUMOylation may act to retard endocytosis, possibly in order to allow full exocytosis of synaptic vesicle contents or proper recovery of synaptic vesicle components. Indeed, we see that the synaptophysin immunostaining is decreased in 3KR-rescue neurones (Fig. S4c). It is not clear how the decreased binding to other SNARE proteins exhibited by SUMOylated Stx1A mediates this ‘brake’ on internalisation. However, one possibility is that non-SUMOylated and SUMOylated Stx1A are located in two distinct pools, with non-SUMOylated Stx1A being involved in SNARE complex formation and membrane fusion, whereas SUMOylated Stx1A is dissociated from its cognate SNARE partners and regulates synaptic vesicle endocytosis under conditions of intense stimulation (for schematic see Fig. 5). Given the proximity of the SUMOylated residues to both the transmembrane domain and the SNARE motif, we assume the SUMOylation of Stx1A could not take place inside the SNARE complex, favouring our model of a separate pool of SUMOylated Stx1A (Fig. 5d). Indeed, modelling studies of the presynapse have shown that many SNARE proteins are located in distinct pools separate from each other^35^.

### Summary

We show that SUMOylation of Stx1A regulates the rate of neurotransmitter vesicle endocytosis but that the rate of exocytosis is not affected. These data suggest that SUMOylation of Stx1A can act as a molecular switch that provides a mechanistic explanation for how Stx1A can mediate distinct functions in synaptic vesicle exocytosis and endocytosis^11^. In conclusion, our results reveal that the activity-dependent SUMOylation of Stx1A provides a novel, flexible and important regulatory mechanism to control neurotransmitter release.

## Methods

### Molecular Biology

Cloning of all constructs was carried out using standard molecular biology methods. For vGlut-pHluorin experiments, vGlut-pHluorin was expressed on the pCAGG vector, contransfected into cells with Stx1 shRNA-Resuce vector. This multifunctional pFIV vector expresses Stx1 shRNA (target sequence **cagaggcagctggagatca**^28^ together with an mCherry-IRES-Stx1A rescue construct (carrying silent point mutations which made Stx1A insensitive to shRNA). pFIV-mCherry was used as a control (containing a ‘stuffer’ fragment after the U6 promoter, meaning these controls did express a non-specific RNA). FM dye experiments used the same knockdown-rescue vector. GST-tagged soluble Stx1A (amino acids 1–266 of *Rattus Norvegicus* Stx1A) was cloned into into pGEX4T-1 vector, and cotransformed with pE1E2-HisSUMO in BL21 (DE3) *E.Coli* for production of SUMOylated Stx1A. All subcloning and mutagenesis reactions were performed according to standard protocols.

### Cell Culture

Embryonic cortical and hippocampal neurones were isolated from E18 Wistar *Rattus Norvergicus* embryos. Brain areas were dissected and trypsinised, before being plated on either PLL-coated 25 mm glass coverslips (for hippocampal cells) or PLL-coated 6-well plates (for cortical cells). Cells were initially plated in plating media (Neurobasal media + 10% horse serum, B27 supplement, 2 mM Glutamax, 1× penicillin/streptomycin), which after 24 hours was replaced with feeding media (Neurobasal media, B27 supplement, 1.2 mM Glutamax, 1× penicillin/streptomycin). For more details, see^36^. For all biochemistry, cortical neurones were used at DIV24–28. For all live cell imaging, hippocampal neurones were used at DIV15. For all live imaging experiments, hippocampal neurons were typically transfected at DIV 11 and imaging experiments were performed four days later. All neuronal transfections were performed using Lipofectamine 2000 (Invitrogen) according to manufacturer’s instructions. For knockdown-rescue experiments, a single multifunctional vector containing Stx1 shRNA and an mCherry-IRES-Stx1A-rescue (either WT or 3KR) cassette was used. Control cells used the same vector without a functional shRNA. For vGlut-pHluorin experiments, cells were cotransfected with this vector and a vGlut-pHluorin vector.

### Biochemistry

All neuronal biochemistry was performed on dissociated rat cortical cultures (Martin *et al.* 2007) at DIV 24–28. For stimulation, cortical cultures were washed once with Earle’s Buffer (25 mM HEPES, 140 mM NaCl, 5 mM KCl, 1.8 mM CaCl_2_, 0.8 mM MgCl_2_, 5 mM glucose, pH 7.4), and equilibrated for 15 minutes at 37 °C in 1 ml Earle’s Buffer. KCl and NMDA were added directly to wells (to final concentrations of 30 mM and 50 μM respectively) and incubated at 37 °C for 30 minutes. Cells were lysed on ice (lysis buffer: 150 mM NaCl, 25 mM HEPES, 1% Triton and 0.1% SDS – pH 7.4, with or without 20 mM N-ethylmaleimide (NEM)), and either mixed with SDS sample buffer for Western blotting or used for immunoprecipitations. For immunoprecipitations, 2 μg of anti Stx1 antibody (HPC-1, Sigma) was bound to Protein G sepharose for 1 hour at 4 °C, after which 200 μg of neuronal lysate was added, followed by another hour at 4 °C. Protein G sepharose was washed and bound protein was eluted with SDS sample buffer. For the HEK293T SUMOylation assay^16^, cells were transfected with Stx1A, Ubc9-Flag and either active SUMO-1-GG-YFP or non-conjugatable SUMO-ΔGG-YFP for 48 hours before lysis on ice in buffer containing 20 nM NEM. The bacterial SUMOylation assay was performed as previously described (Wilkinson *et al.* 2008). Stx1A-SUMO was purified using glutathione sepharose to affinity purify GST-Stx1A, followed by Ni^2+^-NTA agarose to specifically purify SUMOylated Stx1A. Affinity pulldowns from neuronal lysates were performed by binding isolated Stx1A-SUMO to glutathione sepharose beads, incubating for 1 hour at room temperature either with or without recombinant SENP1, followed by incubation with approximately 100 μg of neuronal lysate for 1 hour at 4 °C. Beads were washed and bound proteins eluted with SDS sample buffer before Western blotting.

Quantification of Western blots was performed on Li-Cor Image Studio. All quantified Western blots are presented as the mean ± SEM. Unless otherwise stated, Western blots are representative of at least 3 independent experiments.

Where cropping of blots was performed, the full blots are shown in Fig. S5 with dashed boxes representing the cropped areas.

### Antibodies

The following antibodies were used for Western Blotting: mouse anti Munc18 and anti SNAP-25 (both 1:1000, Cat# 610336 and Cat# 610366, BD Biosciences), mouse anti SUMO1 clone D11 (1:500, Cat# sc-5308, Santa Cruz), rabbit anti VAMP2 (1:10000, Cat# 104 202), mouse anti-ubiquitin (Cell Signalling, Cat# 3936, 1:1000). The following antibodies were used for immunofluorescence: mouse anti SUMO1 clone 21C7 (1:250, Developmental Studies Hybridoma Bank), rabbit anti RIM1 (1:500, Cat# 140 013, Synaptic Systems) and mouse anti HA (1:500, Cat# H3663, Sigma Aldrich), rabbit anti VAMP2 (1:500, cat# 104202, Synaptic Systems), mouse anti Synaptophysin (1:500, cat# 573822, Calbiochem).

### Immunocytochemistry

Immunocytochemistry assays were performed with paraformaldehyde fixation according to standard protocols. Cells were permeabilized in 0.1% Triton except for SUMO-1 staining, in which 20 μg/ml digitonin was used in order to avoid nuclear permeabilisation and thereby reduce the intense SUMO-1 staining normally seen in cell nuclei. All quantification was performed using ImageJ software. In all cases, n = mean of 10–13 ROIs taken from one cell. At least three independent experiments (i.e. on different neuronal cultures on different days) were performed in all cases, with all results normalised to the average of the control values (set to 100%) for each batch of cells. For hippocampal cell KCl stimulation, cells were incubated in HBS (in mM: 140 NaCl, 5 KCl, 1.8 CaCl_2_, 0.8 MgCl_2_, 25 HEPES and 0.9 g/litre glucose - pH 7.4) containing 30 mM KCl for 30 min. For VAMP2 and synaptophysin immunostaining, hippocampal cells were transfected with Lipofectamine 2000 on DIV11 with the knockdown-rescue Syntaxin1a constructs. Cells were then fixed using 4% PFA on DIV15 and permeabilised using Triton X100 (0.1%). 4% BSA was used to block cells and antibody staining was performed with either VAMP2 or Synaptophysin antibodies. Quantification was performed analysing 10 cells per experiment per condition, from 3 different neuronal preparations. 10 puncta were chosen within the transfected cells and the intensity was measured and averaged for each cell. The average intensity of staining for each cell was normalised to the mean staining for the WT-rescue for that neuronal preparation.

All quantified data are presented as the mean ± SEM.

### vGlut-pHluorin Experiments

vGlut-pHluorin experiments were performed as follows: Neurones cotransfected with vGlut-pHuorin, Stx1A knockdown, or knockdown-rescue constructs (see Molecular Biology section for details) were mounted in a Warner stimulation chamber (Harvard Apparatus), connected to a Digitimer Constant Voltage stimulator and a Master-8 pulse train generator. For more details, see^29^. Exocytosis was triggered using electrical field stimulation, using 1 ms pulses (APs) of 50 V. RRP stimulation was triggered by 66 APs at 33 Hz. Reserve pool, or bulk exocytosis, was triggered by 900 APs at 20 Hz. All experiments were performed in HBS with 50 μM D-AP5, 25 μM CNQX with or without 1 μM bafilomycin A. For Fig. 4f,g, two 900 AP 33 Hz stimulations were performed one minute apart. Perfusion with NH_4_Cl (pH 7.4), was used to reveal total levels of vGlut-pHluorin loading of neurones at the end of each experiment. Images were taken at 2 Hz, using a CCD camera with a GFP filter cube. For each cell, 13 ROIs were analysed, with non-responsive ROIs discounted. Cells were taken from at least 4 independent experiments. Data were first normalised to background levels (i.e. ΔF/F_0_), and then expressed as a percentage of fluorescence after NH_4_Cl perfusion (F_max_). Slope analysis of endocytosis was performed by linear regression of the linear phase. Quantified data are presented as the mean ± SEM.

### FM1-43 Experiments

FM1-43 dye loading was carried out as follows: FM1-43 was made up to a stock solution of 5 μg/ml in ddH_2_O and diluted to a working concentrations of 0.5 μg/ml FM1-43 in HBS and kept away from light. Media was aspirated and cells were washed in HBS before incubation with 1 ml of FM1-43 dye for 60 seconds. Synaptic vesicle exocytosis was stimulated through the addition of 45 mM potassium chloride (KCl) for 60 seconds. Nerve terminals were then left to recover in the presence of dye as media was replaced with fresh FM1-43 solution for 60 seconds. To minimise background fluorescence and to allow endocytosed membranes to reform into release-competent vesicles, cells were washed 10 times with HBS (1 ml/minute) before imaging. 25 μM CNQX and 50 μM D-AP5 were present throughout the procedure. For more details, see^34^. For each cell, 13 ROIs were analysed where each ROI encapsulated a single synaptic bouton. Values for knockdown-3KR rescue cells were normalised to knockdown-WT rescue cells in each set of experiments. Cells were taken from at least three independent experiments.

### Statistical Analysis

Graphpad Prism software (Graphpad Inc.) was used for graph plotting and statistical analysis. For comparison of multiple data sets, 1-way ANOVA with Bonferroni’s post-hoc test was used. For comparison of 2 data sets, a 2-tailed Student’s *t*-test was used. Curve fitting for Fig. 3C was performed using Graphpad Prism. All data is reported as mean ± SEM.

## Additional Information

**How to cite this article**: Craig, T. J. *et al.* SUMOylation of Syntaxin1A regulates presynaptic endocytosis. *Sci. Rep.* **5**, 17669; doi: 10.1038/srep17669 (2015).

## Supplementary Material

Supplementary Information

## References

[b1] Sudhof T. C. & Rizo J. Synaptic vesicle exocytosis. Cold Spring Harb. Perspect. Biol. 3, cshperspect.a005637 (2011). 10.1101/cshperspect.a005637 PMC3225952 22026965

[b2] Sudhof T. C. Neurotransmitter release: the last millisecond in the life of a synaptic vesicle. Neuron 80, 675–690 (2013). 24183019 10.1016/j.neuron.2013.10.022 PMC3866025

[b3] Sollner T. *et al.* SNAP receptors implicated in vesicle targeting and fusion. Nature 362, 318–324 (1993). 8455717 10.1038/362318a0

[b4] Risselada H. J. & Grubmuller H. How SNARE molecules mediate membrane fusion: recent insights from molecular simulations. Curr. Opin. Struct. Biol. 22, 187–196 (2012). 22365575 10.1016/j.sbi.2012.01.007

[b5] Han G. A., Malintan N. T., Collins B. M., Meunier F. A. & Sugita S. Munc18-1 as a key regulator of neurosecretion. J. Neurochem. 115, 1–10 (2010). 20681955 10.1111/j.1471-4159.2010.06900.x

[b6] Yang Y. & Calakos N. Munc13-1 is required for presynaptic long-term potentiation. J. Neurosci. 31, 12053–12057 (2011). 21849565 10.1523/JNEUROSCI.2276-11.2011 PMC3201725

[b7] Bacaj T. *et al.* Synaptotagmin-1 and synaptotagmin-7 trigger synchronous and asynchronous phases of neurotransmitter release. Neuron 80, 947–959 (2013). 24267651 10.1016/j.neuron.2013.10.026 PMC3888870

[b8] Deng L., Kaeser P. S., Xu W. & Sudhof T. C. RIM proteins activate vesicle priming by reversing autoinhibitory homodimerization of Munc13. Neuron 69, 317–331 (2011). 21262469 10.1016/j.neuron.2011.01.005 PMC3063404

[b9] Kaeser P. S. *et al.* RIM proteins tether Ca2+ channels to presynaptic active zones via a direct PDZ-domain interaction. Cell 144, 282–295 (2011). 21241895 10.1016/j.cell.2010.12.029 PMC3063406

[b10] Zhang Z. *et al.* The SNARE proteins SNAP25 and synaptobrevin are involved in endocytosis at hippocampal synapses. J. Neurosci. 33, 9169–9175 (2013). 23699527 10.1523/JNEUROSCI.0301-13.2013 PMC3692273

[b11] Xu J. *et al.* SNARE proteins synaptobrevin, SNAP-25, and syntaxin are involved in rapid and slow endocytosis at synapses. Cell Rep. 3, 1414–1421 (2013). 23643538 10.1016/j.celrep.2013.03.010 PMC3672373

[b12] Wilkinson K. A. & Henley J. M. Mechanisms, regulation and consequences of protein SUMOylation. Biochem. J. 428, 133–145 (2010). 20462400 10.1042/BJ20100158 PMC3310159

[b13] Henley J. M., Craig T. J. & Wilkinson K. A. Neuronal SUMOylation: mechanisms, physiology, and roles in neuronal dysfunction. Physiol. Rev. 94, 1249–1285 (2014). 25287864 10.1152/physrev.00008.2014 PMC4187031

[b14] Martin S., Nishimune A., Mellor J. R. & Henley J. M. SUMOylation regulates kainate-receptor-mediated synaptic transmission. Nature 447, 321–325 (2007). 17486098 10.1038/nature05736 PMC3310901

[b15] Shalizi A. *et al.* A calcium-regulated MEF2 sumoylation switch controls postsynaptic differentiation. Science 311, 1012–1017 (2006). 16484498 10.1126/science.1122513

[b16] Craig T. J. *et al.* Homeostatic Synaptic Scaling Is Regulated by Protein SUMOylation. J. Biol. Chem. 287, 22781–22788 (2012). 22582390 10.1074/jbc.M112.356337 PMC3391081

[b17] Jaafari N. *et al.* SUMOylation is required for glycine-induced increases in AMPA receptor surface expression (ChemLTP) in hippocampal neurons. PLoS One 8, e52345, 10.1371/journal.pone.0052345 (2013). 23326329 PMC3543417

[b18] Chamberlain S. E. *et al.* SUMOylation and phosphorylation of GluK2 regulate kainate receptor trafficking and synaptic plasticity. Nat. Neurosci. 15, 845–852 (2012). 22522402 10.1038/nn.3089 PMC3435142

[b19] Feligioni M., Nishimune A. & Henley J. M. Protein SUMOylation modulates calcium influx and glutamate release from presynaptic terminals. Eur. J. Neurosci. 29, 1348–1356 (2009). 19344328 10.1111/j.1460-9568.2009.06692.x PMC3309032

[b20] Girach F., Craig T. J., Rocca D. L. & Henley J. M. RIM1alpha SUMOylation is required for fast synaptic vesicle exocytosis. Cell Rep. 5, 1294–1301 (2013). 24290762 10.1016/j.celrep.2013.10.039 PMC3898736

[b21] Dai X. Q. *et al.* SUMOylation regulates insulin exocytosis downstream of secretory granule docking in rodents and humans. Diabetes 60, 838–847 (2011). 21266332 10.2337/db10-0440 PMC3046844

[b22] Xue Y., Zhou F., Fu C., Xu Y. & Yao X. SUMOsp: a web server for sumoylation site prediction. Nucleic Acids Res. 34, W254–257 (2006). 16845005 10.1093/nar/gkl207 PMC1538802

[b23] Hay R. T. SUMO: a history of modification. Mol. Cell 18, 1–12 (2005). 15808504 10.1016/j.molcel.2005.03.012

[b24] Chin L. S., Vavalle J. P. & Li L. Staring, a novel E3 ubiquitin-protein ligase that targets syntaxin 1 for degradation. J. Biol. Chem. 277, 35071–35079 (2002). 12121982 10.1074/jbc.M203300200

[b25] Na C. H. *et al.* Synaptic protein ubiquitination in rat brain revealed by antibody-based ubiquitome analysis. J Proteome Res. Sep 7;11(9):4722–32. 10.1021 (2012). 22871113 10.1021/pr300536k PMC3443409

[b26] Sudhof T. C. & Rothman J. E. Membrane fusion: grappling with SNARE and SM proteins. Science 323, 474–477 (2009). 19164740 10.1126/science.1161748 PMC3736821

[b27] Smyth A. M., Duncan R. R. & Rickman C. Munc18-1 and syntaxin1: unraveling the interactions between the dynamic duo. Cell Mol. Neurobiol. 30, 1309–1313 (2010). 21046456 10.1007/s10571-010-9581-1 PMC11498833

[b28] Zhou P. *et al.* Syntaxin-1 N-peptide and Habc-domain perform distinct essential functions in synaptic vesicle fusion. EMBO J. 32, 159–171 (2013). 23188083 10.1038/emboj.2012.307 PMC3545302

[b29] Burrone J., Li Z. & Murthy V. N. Studying vesicle cycling in presynaptic terminals using the genetically encoded probe synaptopHluorin. Nat. Protoc. 1, 2970–2978 (2006). 17406557 10.1038/nprot.2006.449

[b30] Sankaranarayanan S. & Ryan T. A. Real-time measurements of vesicle-SNARE recycling in synapses of the central nervous system. Nat. Cell Biol. 2, 197–204 (2000). 10783237 10.1038/35008615

[b31] Wu W., Xu J., Wu X. S. & Wu L. G. Activity-dependent acceleration of endocytosis at a central synapse. J. Neurosci. 25, 11676–11683 (2005). 16354926 10.1523/JNEUROSCI.2972-05.2005 PMC1378116

[b32] Granseth B. & Lagnado L. The role of endocytosis in regulating the strength of hippocampal synapses. J.Physiol. 586, 5969–5982 (2008). 19001048 10.1113/jphysiol.2008.159715 PMC2655433

[b33] Mishima T. *et al.* Syntaxin 1B, but not syntaxin 1A, is necessary for the regulation of synaptic vesicle exocytosis and of the readily releasable pool at central synapses. PLoS One 9, e90004, 10.1371/journal.pone.0090004 (2014). 24587181 PMC3938564

[b34] Gaffield M. A. & Betz W. J. Imaging synaptic vesicle exocytosis and endocytosis with FM dyes. Nat. Protoc. 1, 2916–2921 (2006). 17406552 10.1038/nprot.2006.476

[b35] Wilhelm B. G. *et al.* Composition of isolated synaptic boutons reveals the amounts of vesicle trafficking proteins. Science 344, 1023–1028 (2014). 24876496 10.1126/science.1252884

[b36] Martin S. & Henley J. M. Activity-dependent endocytic sorting of kainate receptors to recycling or degradation pathways. EMBO J. 23, 4749–4759 (2004). 15549132 10.1038/sj.emboj.7600483 PMC535095

