# Supplementary material for: SUMOylation of Syntaxin1A regulates presynaptic endocytosis
Source: Sci Rep. 2015 Dec 4;5:17669. doi: 10.1038/srep17669 (PMC4669484; doi:10.1038/srep17669)
Supplement: Supplementary Information [file srep17669-s1.pdf]

# SUMOylation of Syntaxin1A regulates presynaptic endocytosis

**Tim J. Craig, Dina Anderson, Ashley J. Evans Fatima Girach and Jeremy M. Henley**

School of Biochemistry, Medical Sciences Building, University of Bristol, University Walk, Bristol, BS8 1TD, UK

## Supplementary Information

# Figure S1 – Mutation of individual sites preserves Stx1A SUMOylation

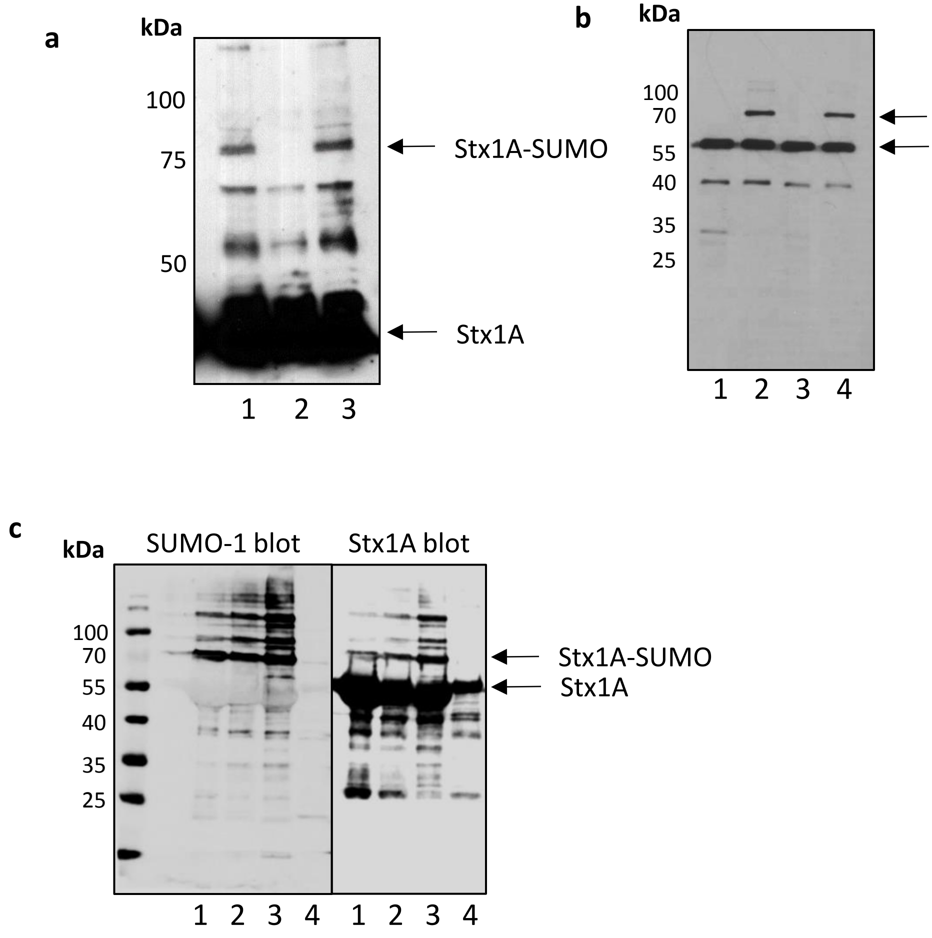

# Figure S2 – 3KR mutation displays normal binding, distribution and does not affect ubiquitination

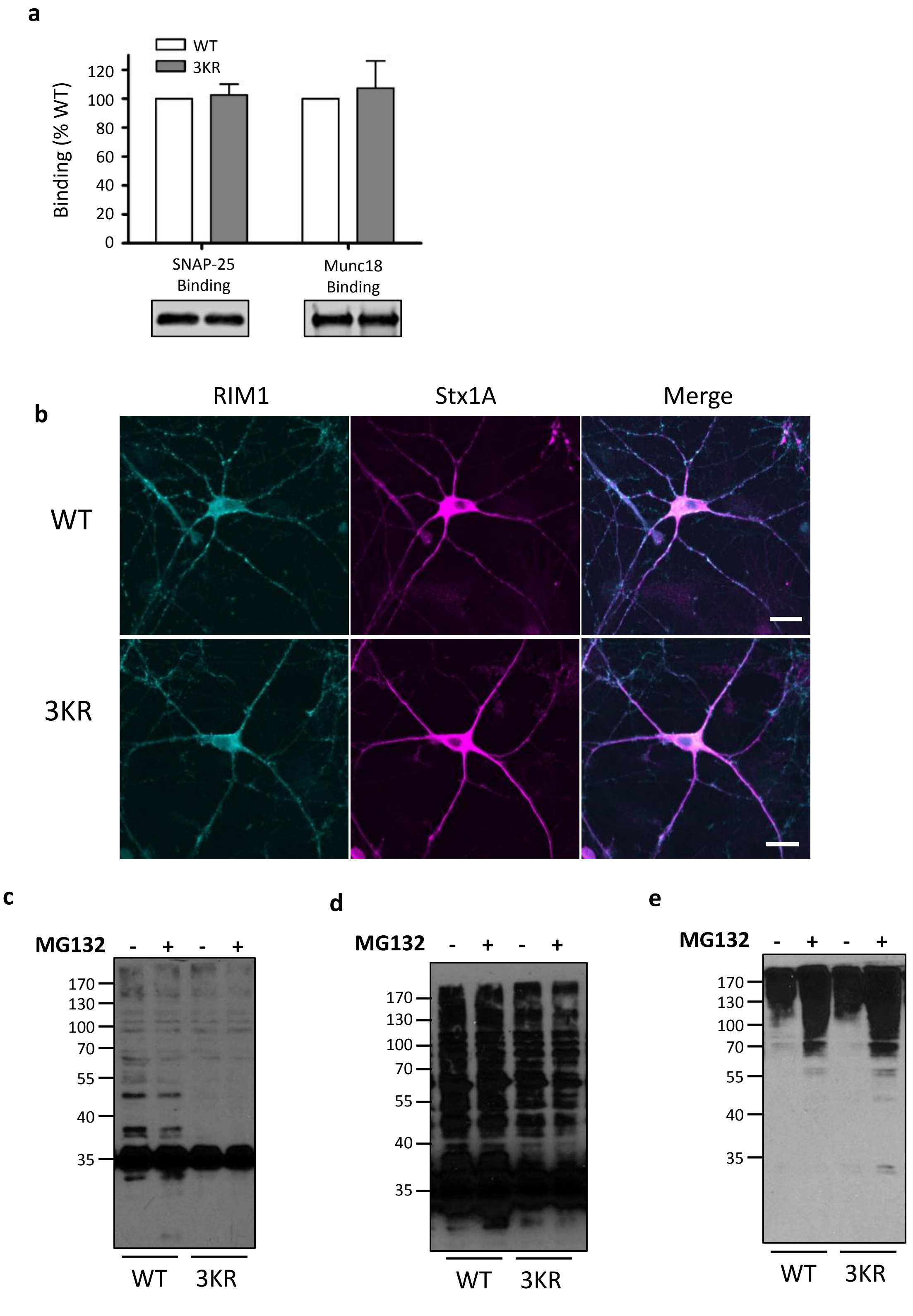

# Figure S3 – Efficacy of Stx1 shRNA

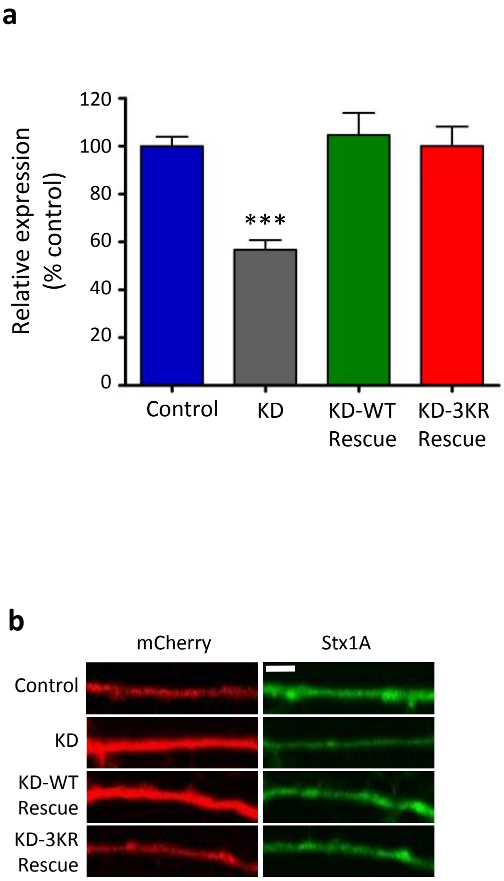

# Figure S4 – The size of the SV pool is unchanged in 3KR rescue neurones

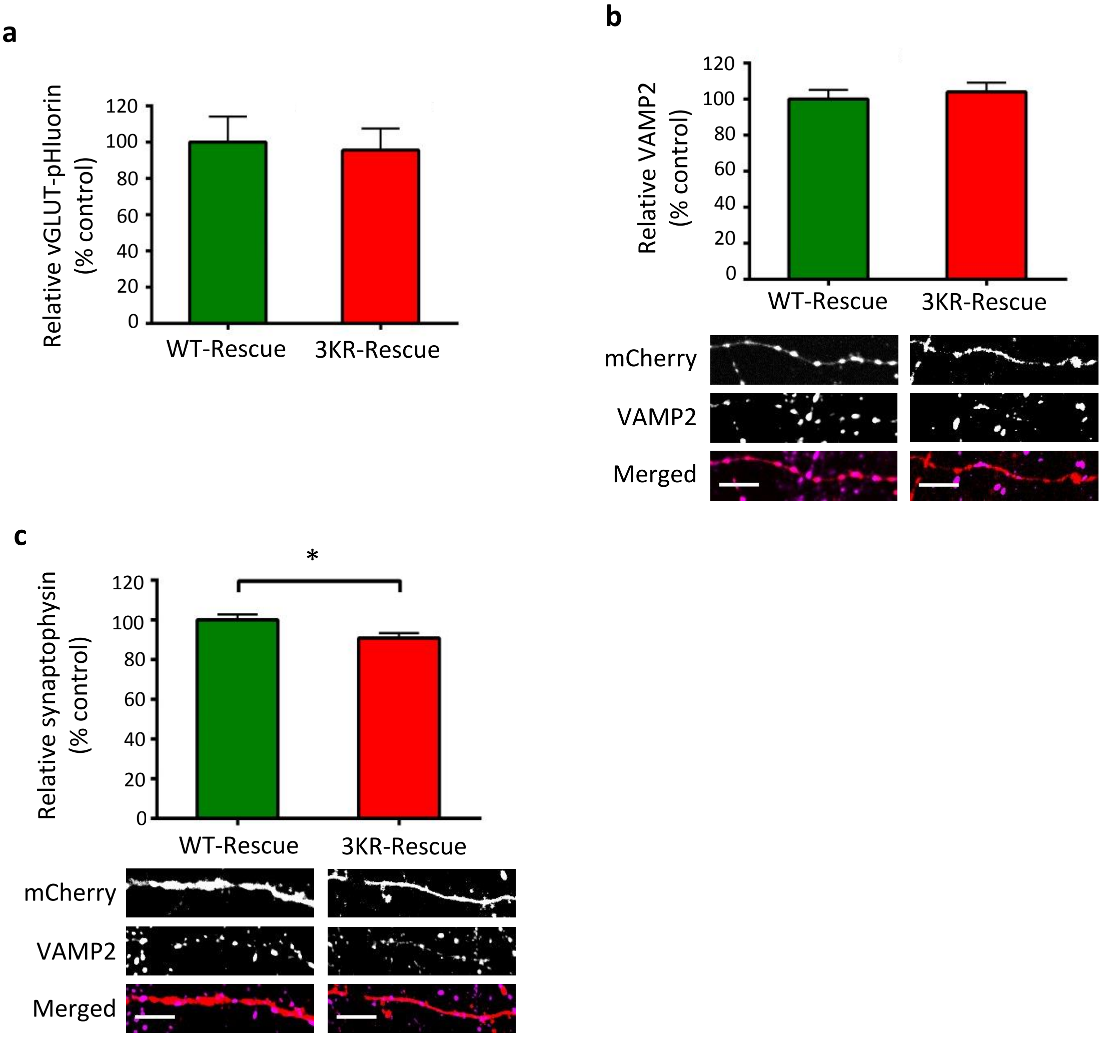

# Figure S5– Uncropped blots

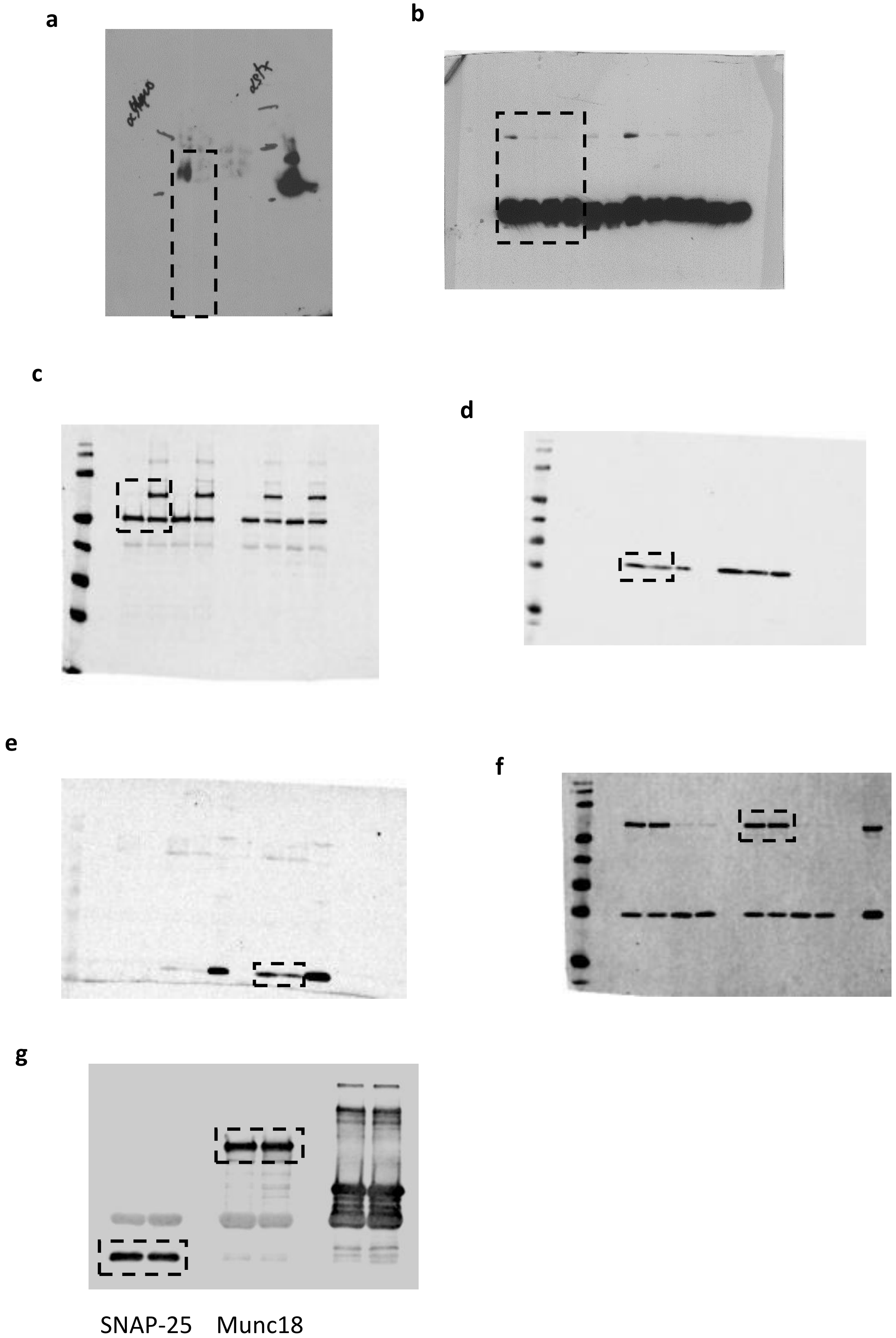

# Supplementary Figure Titles and Legends

## Figure S1– Mutation of individual sites preserves Stx1A SUMOylation

**a:** HEK293 cells SUMOylation assay of WT and K204R Stx1A with YFP-SUMO1. Cells transfected with 1: Stx1A WT, Ubc9 + YFP-SUMO1, 2: Stx1A WT, Ubc9 + YFP-SUMO1 ΔGG, 3: Stx1A K204R, Ubc9 + YFP-SUMO1.

**b:** *E.Coli* SUMOylation assay of WT + K204R Stx1A. Lane 1: Stx1A WT + SENP1 treatment, 2: Stx1A WT, 3: Stx1A K204R + SENP1 treatment, 4: Stx1A K204R.

**c:** *E.Coli* SUMOylation assay of K252R, K253R and K256R Stx1A. Lane 1: WT Stx1A, 2: K252R Stx1A, 3: K253R Stx1A, 4: K256R Stx1A. Note that although SUMOylation appears much lower for K256R, this is due to a greatly decreased expression level (see Stx1A blot).

## Figure S2 – 3KR mutation displays normal binding, distribution and does not affect ubiquitination

**a:** Affinity pulldown from cortical neuronal lysate using GST-Stx1A 3KR, blotted to SNAP-25 and Munc18. Graph shows quantification (n=4), normalised to the wild-type binding, with no significant difference in binding detected. Blots below are from representative experiments.

**b:** Immunostaining of Stx1A-HA transfected neurones, showing similar distribution of WT and 3KR Stx1A. Neurones were immunostained with anti-HA (magenta) and co-stained with anti-RIM1 (cyan). Scale bar = 20 µm.

**c-e:** Lysates of HEK cells transfected with Stx1A WT or 3KR, treated with 20 µM MG132 for 4 hours where indicated. **c:** Stx1A blot, **d:** long exposure Stx1A blot, **e:** Ubiquitin blot.

## Figure S3 – Efficacy of Stx1 shRNA

**a:** Quantification of Stx1A levels detected by immunostaining with HPC-1 of hippocampal neurones transfected with Stx1 shRNA and rescue constructs. Values are normalised to the average control values for each set of experiments. Control n=25, KD n=20, KD-WT rescue n=17, KD-3KR rescue n=15, from at least 3 separate experiments. ***, $p < 0.001$ (1 way ANOVA).

**b:** Representative images of neuronal processes used for quantification. mCherry is used as a transfection marker. Scale bar = 10 µm.

## Figure S4 – 3KR mutation does not change the synaptic vesicle pool size

**a:** Quantification of total vGLUT-pHluorin fluorescence, taken as an average of the signal at 10 time-points following $NH_4Cl$ treatment. These data are from the same cells as the data in **Fig3a,b,c**. 3KR-rescue signals (n=16) are averaged to the mean of the WT-rescue (n=13) signals.

**b:** Confocal imaging of Syntaxin1a knockdown-rescue with either WT or 3KR mutant, with VAMP2 used as a synaptic vesicle pool marker. Graph: quantification of VAMP2 signals (n=30 for both). Representative mCherry, VAMP2 and merged images are shown below quantification. Scale bar = 10µm.

**c:** Confocal imaging of Syntaxin1a knockdown-rescue with either WT or 3KR mutant, with synaptophysin used as a synaptic vesicle pool marker. Graph: quantification of VAMP2 signals (n=30 for both). *, $p < 0.05$. Representative mCherry, VAMP2 and merged images are shown below quantification. Scale bar = 10µm.

## Figure S5 – Uncropped blots

**a:** Full blot from Fig 1b. Cropped area is indicated with a dashed box.

**b:** Full blot from Fig 1g. Cropped area is indicated with a dashed box.

**c:** Full Stx1a blot from Fig 2b. Cropped area is indicated with a dashed box.

**d:** Full SNAP-25 blot from Fig 2b. Cropped area is indicated with a dashed box.

**e:** Full VAMP2 blot from Fig 2b. Cropped area is indicated with a dashed box.

**f:** Full Munc18 blot from Fig 2b. Cropped area is indicated with a dashed box.

**g:** Full Munc18/SNAP-25 blot from Fig S1c. Cropped area is indicated with a dashed box.
